# Optimizing and Benchmarking Machine Learning and Traditional Synaptic Event Detection Pipelines in Neurophysiology Experiments

**DOI:** 10.1523/ENEURO.0410-25.2026

**Published:** 2026-04-29

**Authors:** Joshua P. Sevigny, Sean Schrank, Rachel M. Donka, Oscar D. Aguilar, N. Ika Yunus, Mikaela R. Valchinova, Zach Fyke, Joseph D. Zak, Jamie D. Roitman, Dennis R. Sparta

**Affiliations:** ^1^Department of Psychology, University of Illinois at Chicago, Chicago, Illinois 60607; ^2^Graduate Program in Neuroscience, University of Illinois at Chicago, Chicago, Illinois 60607; ^3^Department of Biology, University of Illinois at Chicago, Chicago, Illinois 60607

**Keywords:** electrophysiology, machine learning, synaptic physiology

## Abstract

Synaptic physiology experiments are fundamental to neuroscience research. Consequently, accurate detection of synaptic currents is crucial for conducting high-quality experiments. Traditionally, detecting inhibitory and excitatory postsynaptic currents (sIPSCs/sEPSCs) relied on hand-counting individual events. Although sEPSCs and sIPSCs are clear to the trained eye, hand analysis is time and labor intensive. Recent advances in applied machine learning promise faster, superior event detectors that may improve data quality and reduce or even completely negate the need for hand curation. While many strategies for sIPSC and sEPSC detection exist, rarely have they been quantitatively compared for accuracy within an experiment. Our study aims to establish practical ground-truth event detection in a large experimental dataset through meticulous hand counting and to assess variance in detection results across different laboratories, analysis techniques, and cell types. Using thoroughly hand-counted data as our ground-truth comparison, we benchmark current popular detection methods, including a modern supervised deep learning approach. Our results suggest that current analysis strategies vary widely in their results and that a supervised machine learning approach rivals manual event counting performed by expert electrophysiologists better than other automated approaches.

## Significance Statement

Our study aims to measure interlab variability and to benchmark specific inhibitory and excitatory synaptic event detection techniques, including hand counting and the main automated approaches used in the field of slice electrophysiology.

## Introduction

Electrochemical communication via synaptic transmission forms the basis for how the brain functions, and our ability to understand this process is dependent upon our ability to measure it (Hodgkin and Huxley, 1952b; Südhof and Malenka, 2008; Kavalali, 2015; Appelbaum et al., 2023). Recording subthreshold channel activation with whole-cell patch-clamp electrophysiology provides insight into the fundamental mechanisms of how neurons communicate (McKinney et al., 1999; Glasgow et al., 2019), and the fidelity of these measurements is therefore crucial to understanding the brain. By analyzing both the size of the currents and the frequency of spontaneous excitatory and inhibitory (sEPSC and sIPSC) events, an experimenter can observe one of the functional components of neuronal circuitry (Johnston and Wu, 1995; O’Neill et al., 2025). Like many modern neuroscience techniques, the roots of the technique itself are historic (Hodgkin and Huxley, 1952b), but over time both the physical technique and the methods by which data is analyzed have evolved significantly. The replicability and reproducibility crisis is often discussed in science and is usually centered around statistical power in experiments. Another contributing component of the crisis, however, is the growing use of a wide array of computational approaches for data collection and processing. A recent finding highlights potential fundamental limitations with spontaneous synaptic event measurement and interpretation. Greger and Watson find that synaptic event detection is highly sensitive to changes in detection thresholds and noise characteristics even under well-controlled conditions (Greger and Watson, 2024). While computational models promise answers to complex problems, the models and methods themselves must be thoroughly vetted.

Whole-cell patch-clamp electrophysiology is a powerful tool, albeit complex and costly in terms of equipment and highly trained personnel time (Neher and Sakmann, 1976). Concomitantly, data collection and analysis are not trivial. While the general shape of an sEPSC or sIPSC is identifiable in an electrophysiology trace by a trained professional, hand-scoring a 3 min gap-free recording may take as long as an hour or more, resulting in an analysis bottleneck on experimental throughput (Wuarin and Dudek, 1993). Subsequently, automated approaches including baseline threshold techniques, templating approaches, and beyond (Clements and Bekkers, 1997; Kudoh and Taguchi, 2002; Shi et al., 2010; Pernía-Andrade et al., 2012; Kim et al., 2021; Zhang et al., 2021; Wang et al., 2024) have been sought to relieve this analysis bottleneck, which has resulted in a plethora of hybrid analysis approaches (Ankri et al., 1994), which are not routinely subjected to validation against thoroughly hand-counted data. In other neuroscience disciplines, analysis pipeline comparison studies are becoming increasingly common especially in the functional imaging domain (Bhagwat et al., 2021; Luppi et al., 2024) and are vital for maintaining integrity and reproducibility as techniques and methods evolve.

Most recently, the generation of event detection models using deep learning (DL) has offered a potential solution (O’Neill et al., 2025). While the goal of detecting “real” events remains the same, DL promises a practical, less computationally rigid method of event detection that has the potential to parse events and inconsistent noise more effectively, possibly even replicating the quality of manual counting (O’Neill et al., 2025). While DL models lack the rigid consistency of simple thresholding techniques, their more flexible nature may allow them to better avoid labeling noise. The ability to train a model with a substantial training dataset including events with varying degrees of clarity as well as a vast spectrum of noise traces could theoretically produce a model nuanced enough to discriminate between real events and noise, even when recording conditions are not ideal, and noise is inconsistent. For example, a DL framework (miniML) for generating such models has been outlined by O’Neill et al. Using their training framework, they generate a model capable of sEPSC detection and a transfer-learning technique for adapting it to specific datasets. While their ML implementation is sophisticated and can clearly detect sEPSCs, their validation process uses clean traces and lacks testing in an experimental context. Also notably, the “ground truth” they use for benchmarking the accuracy of their own analysis is synthesized through a template/threshold technique where the “correct” answers are defined by the template used to generate the synthetic trace which we feel may not adequately represent a true use case. In summary, their final model is trained on events detected by a semiautomated templating technique and benchmarked against simulated ground-truth data generated with the same event template. While this approach is certainly viable and useful, it could limit the quality of the final model's ability to translate to real biology. As such, it is possible the performance of the model and the benchmarks applied may reflect internal consistency more so than biological accuracy.

Considering all of the different analysis methods, what should be considered ground truth for benchmarking purposes? In any study which seeks to evaluate the ability, accuracy, and precision of a technique, there must be a standard by which it is measured. While a perfectly objective ground truth does not exist, we argue that a thorough hand count by a seasoned electrophysiologist, while excruciating to conduct, is the highest quality possible count, and the best representation of the field's consensus on what “real events” look like (Hodgkin and Huxley, 1952a; Wuarin and Dudek, 1993; Johnston and Wu, 1995). Thus, rather than simply presenting our findings as differences in variance between currently used methods, we have chosen to meticulously hand count our entire dataset as a practical ground-truth comparison. This choice reflects careful consideration for the history of event quantitation within the field and our own internal review. Using this as a guide, we have built a framework for evaluating automated or semiautomated detectors against our practical ground-truth detection. Here, we compare popular detection techniques in a fully powered pharmacology experiment.

While internal validations of specific techniques have been demonstrated, a broad comparison of sIPSC and sEPSC detectors in a large, real, experimental dataset that has been thoroughly hand counted has not been attempted. We attempt to compare current real-world laboratory analysis pipelines using the same dataset to derive a baseline variance between detection methods currently used and respected in the field. We also generated a separate, extensive dataset containing thousands of excitatory and inhibitory event and noise segments from a variety of cell types and brain regions and hand curated a massive, representative training dataset. Building upon miniML's already robust training framework (O’Neill et al., 2025), we used this training data to generate sIPSC and sEPSC detection models reflecting the variance in recording data specific to our lab, to compare with our other traditional approaches. Finally, to properly benchmark these models and the other commonly used detection techniques, we collected and hand-scored a separate pharmacology study in order to investigate not only which detectors find the closest agreement to our practical ground-truth detection but also to compare the statistical outcomes found by each respective detection method.

Cumulatively our results demonstrate that beyond the physical limits of signal-to-noise ratio inherent in the technique, detector-dependent variability across laboratories and approaches introduces even more noise into the equation, highlighting the need for practical benchmarking of detection approaches. This work provides a quantitative comparison of commonly used detection methods, as well as a modern machine learning approach with the goal of accurately representing variance between detection methods using an application-based approach. We believe this work, and future work like it will help shed light on a path forward for increasing the reliability and reproducibility of synaptic event detection.

## Materials and Methods

### Ex vivo whole-cell patch-clamp electrophysiology protocol for training dataset and pharmacology test dataset

Animals were deeply anesthetized by isoflurane and then transcardially perfused with ice-cold oxygenated aCSF (in mM): 130 NaCl, 2.5 KCl, 2.0 CaCl_2_, 1.2 MgSO_4_, 1.25 KH_2_PO_4_, 25 NaHCO_3_, and 10 dextrose (305–310 mOsm). For mice used in the pharmacology study, saline or ethanol (2 g/kg) was intraperitoneally injected 1 h prior to perfusion. Brains were then extracted and mounted on a Leica VTS1200 Bath Vibratome (filled with cold, oxygenated aCSF), and 240 µm coronal slices were prepared where they were then transferred to recovery chambers with warmed (33°C) aCSF and allowed to recover for 20–30 min. Recordings in the pharmacology study were taken from fluorescent CRF+ cells from the central amygdala (CeA). Patch-clamp recordings were made using a cesium methanesulfonate-based internal solution containing the following (in mM): 135 CsMeSO_3_, 10 KCl, 1 MgCl_2_, 0.2 EGTA, 4 Na_2_ATP, and 0.3 Na_2_GTP, adjusted to pH 7.3. After establishing a stable whole-cell configuration, gap-free recordings were made in voltage-clamp holding at −70 mV for 3′ (sEPSC), then the applied hold was changed to +10 mV for 1′ or until holding current was stable. A gap-free recording was then again made +10 mV for 3′ (sIPSC). Data was collected using a MultiClamp 700B amplifier digitized at 20 kHz with an Axon Digidata 1550B. RA was monitored throughout the recording and cells exceeding RA of 25 megaohms were not used in analysis. Data were collected using pClamp 11.2.

### Ex vivo whole-cell patch-clamp electrophysiology protocol for external laboratory validation dataset

Acute horizontal sections of the olfactory bulb were extracted from C57BL/6J female P51 mice. Animals were anesthetized using isoflurane and decapitated after perfusion with chilled artificial CSF (aCSF). Horizontal sections (300 μM) were sliced using a vibrating microtome (Leica VT 1000S) in aCSF composed of the following (in mM): 105 choline chloride, 20 glucose, 24 NaHCO_3_, 2.5 KCl, 8 MgCl_2_, 0.5 CaCl_2_, 5 sodium ascorbate, 3 sodium pyruvate, and 1.25 NaH_2_PO_4_. Slices were incubated in a similar aCSF solution containing the following (in mM): 115 NaCl, 20 glucose, 25 NaHCO_3_, 2.5 KCl, 2 MgCl_2_, 1 CaCl_2_, and 1.25 NaH_2_PO_4_, equilibrated in carbogen (95% O_2_ and 5% CO_2_) at 34°C for 30 min. Room temperature slices were transferred to a recording chamber with an upright Olympus microscope under 4×, 10×, and 40× objectives, respectively.

For whole-cell patch-clamp recordings, glass recording pipettes (4 inch thin wall gl 1.0 OD/.75ID, World Precision Instruments) were pulled to a resistance of 4–8 MOhm on a Narishige PC-100. Internal solution for voltage-clamp recordings was composed of a cesium methanesulfonate solution: 125 CsMeSO_3_, 2 MgCl_2_, 0.025 CaCl_2_, 1 EGTA, 2 Na_2_ATP, 0.5 Na_2_GTP, 10 HEPES, and supplemented with 594 hydrazide dye for visualization (21 μM). Voltage-clamp recordings at −70 and 0 mV were collected at 36°C using a MultiClamp 700B amplifier and digitized at 10 kHz with an Axon Digidata 1550B. Synaptic currents were filtered with a Bessel filter set at 1.8 kHz. Data were collected using pClamp 11.2.

### Collaborator laboratory spontaneous event detection methods

**Figure 1.**
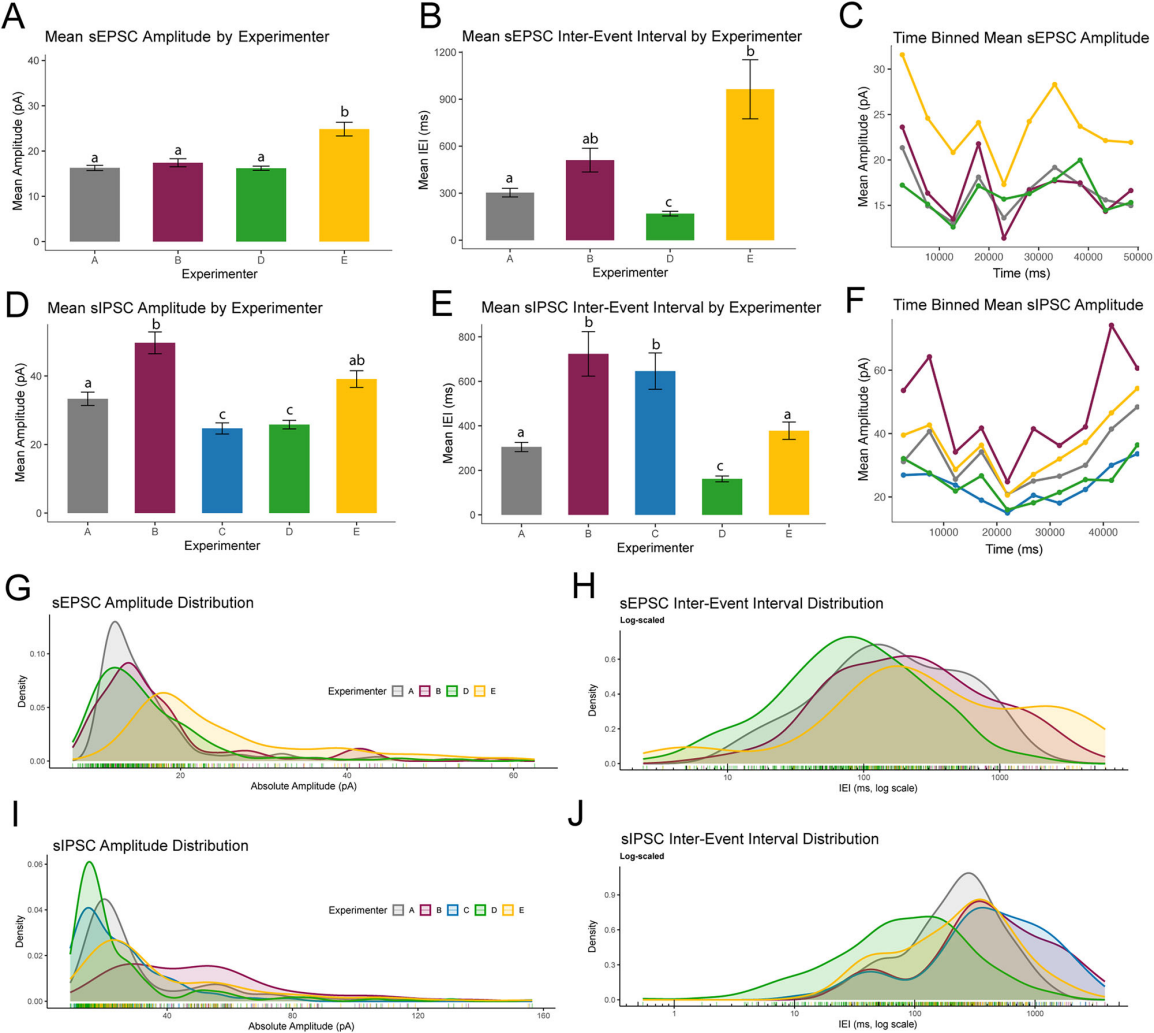
Interlab variability in synaptic event quantification from traditional analyses. Identical sIPSC and sEPSC traces analyzed by different electrophysiologists/laboratories. ***A***, Mean sEPSC event amplitude by experimenter. ***B***, Mean sEPSC IEI variation across experimenters. ***C***, Amplitude variation throughout the trace for each experimenter. ***D***, Mean sIPSC amplitude by experimenter. ***E***, Mean sIPSC IEI variation across experimenters. ***F***, Amplitude variation over entire trace duration for each experimenter. ***G***, ***H***, Overlaid histograms showing variation of detected sEPSC amplitude and IEI distributions between experimenters. ***I–J***, Overlaid histograms showing variation of detected sIPSC amplitude and IEI distributions between experimenters. Error bars represent SEM. Full statistical reporting provided in Extended Data Figure 1-1.

To assess the general variance between labs in synaptic event detection, we sent the same sEPSC and sIPSC traces to collaborating electrophysiology labs for analysis. Collaborating laboratories were not constrained in methodological approach to spontaneous event detection so that an assessment of the field could be accurately made (Fig. 1). Without identifying each lab, their approaches are discussed below.

10.1523/ENEURO.0410-25.2026.f1-1 Figure 1-1 **Statistical Results** Table showing Welch’s ANOVA results and relevant post-hoc, Games-Howell corrected t-tests comparing sEPSC and sIPSC amplitude and IEI between experimenters. Download Figure 1-1, CSV file.

#### Hand count

Two labs reportedly used a base event threshold set to 5 pA, and then all events reported were manually verified for the length of the recording using the software MiniAnalysis, one using pClamp Clampfit 11.2.

#### Automatic threshold

One collaborating lab used an automatic event detection in pClamp Clampfit 11.2 based on a threshold setting of 3 standard deviations from the noise band calculated after a 1,000 Hz Bessel filter. Events were automatically collected and not manually curated.

#### Automatic threshold

One collaborating lab used an automatic event detection within AxoGraph using a template and threshold method where a portion of events were collected with a flat threshold of +30 pA for sIPSC or a −20 pA threshold for sEPSCs, and then templates were made from averaged events captured. This created template then was used to score events within the recording with a detection threshold set at 5× standard deviations beyond the noise band. Events with rise times <30 mcs were excluded.

#### Automated template threshold

One collaborating lab reported that a threshold was applied to set the minimum amplitude of detected events >5 pA, with the automated current detection criterion set as a correlation cutoff at 0.6.

It should be noted that one collaborating lab (Experimenter C) was unable to analyze our sEPSC trace but did provide analysis of the sIPSC trace given.

### Event detection approaches for benchmarking in experimental pharmacology dataset

To provide a comprehensive assessment of the state of spontaneous event measurement, we conducted five common methods of data analysis: program assisted hand curation (HD2), threshold-based automated analysis (BTD), threshold and templated based automated analysis (TTD), custom mean absolute deviation based peak detection (MAD), and a custom trained deep learning model (MLD; Table 1).

**Table 1.** Detection methods legend

Detection method	Detection type	Description
HD1/HD2	Primary/secondary hand count	Events individually scored manually by experimenters
BTD	Baseline threshold detection	Events scored based on crossing an amplitude threshold relative to baseline noise
TTD	Threshold template detection	Detect events by correlating data with predefined template waveform
MAD	Median absolute detection	Detect events based on mean absolute deviations from baseline noise
MLD	Machine learning detection	Detect events using deep learning model trained on large hand curated dataset

Legend showing each detection method and its corresponding abbreviation.

We chose to test detectors in a highly constrained ethanol study familiar to our lab in order to best model a modern pharmacology experiment. CeA CRF+ cells are a relatively homogenous population of neurons considered important for ethanol seeking. This dataset is highly representative of current work in our lab and was therefore ideal for benchmarking detectors in a relevant experiment in the context of our work. Each analysis was conducted on the same experimental dataset containing recordings from CRF+ CeA cells in 8–14-week-old male and female mice [C57BL/6J background; Crh-IRES-Cre × B6.Cg-Gt(ROSA)26Sortm9(CAG-tdTomato)Hze/J] across two experimental groups: IP ethanol (sIPSC *N* = 13 cells, sEPSC *N* = 14 cells) and IP saline (sIPSC *N* = 11 cells, sEPSC *N* = 9 cells; Extended Data Fig. 2-2). Adult mice were administered intraperitoneal (IP) injections of either 2 g/kg ethanol or saline, and whole-cell patch-clamp recordings were collected from acute brain slices prepared 60 min post-injection. The 2 g/kg dose has been shown to produce intoxicating blood alcohol concentrations (over 100 mg/dl) 60 min postacute intraperitoneal injection (Lopez et al., 2012). Inhibitory and excitatory currents were recorded under voltage clamp from CRF+ neurons in the central amygdala.

### Hand detection strategy

The hand detection method (HD1, HD2) involved the experimenter visually analyzing the recording and manually identifying spontaneous events in either Easy Electrophysiology v. 2.6.3 or pClamp Clampfit 11.2 using a basic threshold filter to screen out noise. A standard Gaussian filter was applied prior to analysis. Experimenter set threshold outside of the noise band of the recording and then manually selected which events with peak amplitudes over threshold were true biological signal versus that of electrical noise.

### Threshold-based automated detection strategy

The threshold-based automated analysis was performed by previewing a 5–10 s section of the beginning of the recording and then applying an appropriate amplitude event threshold based upon the size of the noise band for the duration of the recording. A standard Gaussian filter was applied prior to analysis. For recordings with considerable drift, threshold analysis was segmented, and new thresholds were established per segment. pClamp Clampfit 11.2 was used to conduct the baseline threshold detection.

### Template-based automated detection strategy

The threshold and template analysis was performed similarly to the threshold analysis. A standard Gaussian filter was applied prior to analysis. Additionally, this approach included the creation of a template for three representative events per recording. In this way, events crossing the amplitude threshold were then screened against the template bank and characteristics were automatically applied for inclusion into the dataset. Easy Electrophysiology v. 2.6.3 was used to conduct the template-based detection approach.

### Mean absolute deviation detection strategy

To apply threshold-based detection of events, sIPSC and sEPSC recordings were filtered with a third-order bandpass Butterworth filter (high-pass cutoff of 0.05 Hz, low-pass cutoff of 50 Hz). To approximate the noise band for each recording, the raw recordings were passed through a high-pass third-order Butterworth filter (high-pass cutoff of 100 Hz). The mean absolute deviation (MAD) of the filtered recordings and generated noise band were determined for each recording. Threshold parameters were determined based on comparison of threshold detected events to hand-counted events in three recordings of each type to maximize accuracy.

For sIPSC recordings, the event inclusion threshold was set at the median of the filtered recording plus 3.6 times the MAD of the noise band. For sEPSC recordings, the event inclusion threshold was set at the median of the filtered recording minus 2.5 times the MAD of the noise band. All local maxima in the filtered recording were identified, and amplitude was determined as peak maximum minus prepeak baseline (minimum value from 15 to 6 ms before the peak). Events that surpassed the threshold were included and amplitudes were determined in both the filtered and unfiltered recording for analysis. All MAD analysis was conducted in MATLAB v. 9.13.0 (Donka et al., 2025).

### Deep learning training data generation and strategy

Our deep learning models were trained on a vast collection (*N* = 54 animals/130 cells) of sEPSC and sIPSC slice electrophysiology recordings from our laboratory in male and female C57BL/6J background mice, Sprague Dawley rats, and Long–Evans rats (Extended Data Fig. 2-2). To best represent the particularities of data generated by our recording setup to the model, we strove to compile as diverse a dataset as possible within the confines of our own lab's capacities and scope. Recordings were collected using the same basic electrophysiology protocol as the pharmacology dataset, from a variety of regions and cell types including dopamine neurons in the ventral tegmental area as well as CRF and GABAergic neurons from the central amygdala and ventral bed nucleus of the stria terminalis. While we cannot claim this dataset is representative of synaptic activity from all species, regions, and cell types, our goal was to capture as much variability in synaptic events and noise signatures as possible within the confines of our own recording environment (Extended Data Fig. 2-2). Our final sIPSC dataset contained (15,170) hand-verified, individual event and noise segments ranging in size, shape, and clarity (Extended Data Figs. 2-1, 2-2). Our final sEPSC training dataset contained (36,316) hand-verified, individual event and noise segments ranging in size, shape, and clarity. For both models, event to noise ratio was held at 1.0.

As previously mentioned, while variance in synaptic event kinetics is high, it pales in comparison to the wide variety of noise signatures possible in synaptic recordings. To address this, it was important not only to include an equal number of noise traces, but also to ensure those noise traces were representative of the wide variety of noise signatures possible at least in our recording environment. We recorded an enormous amount of real-world background noise, purposefully generating common and uncommon types of noise possible in whole-cell recordings (Extended Data Fig. 2-1). These noise traces were then injected into our training datasets so that the wide variety of noise signatures learned by the neural network would better reflect the diversity of noise possible. We believe our focus on purposefully including diverse noise signatures is key to the success of the model and suggest that others creating their own models may want to use our real noise library or generate noise relevant to their own recording environment to bolster their own training datasets.

### Model training

**Figure 2.**
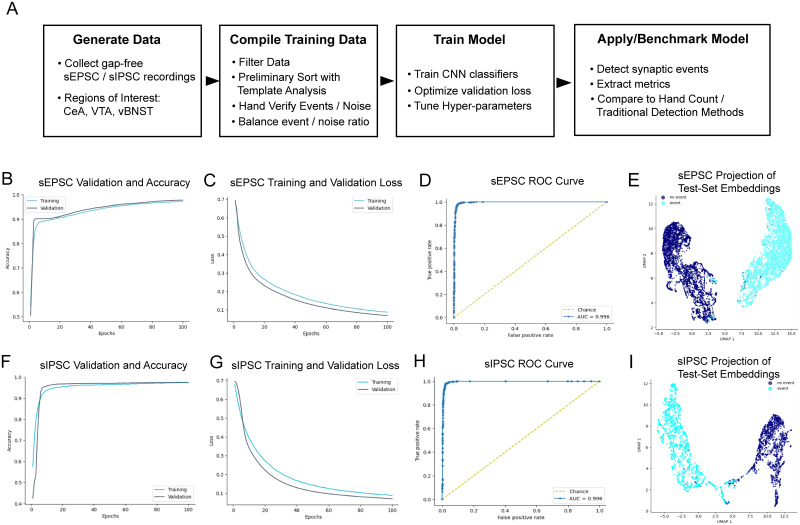
Deep learning model training workflow and training validation. ***A***, Deep learning training and detection workflow. ***B***, Training and validation accuracy per epoch, showing successful training of sEPSC-detecting deep learning model. ***C***, Training and validation loss per epoch for training of sEPSC model. ***D***, ROC curve generated testing model performance on withheld sEPSC training data, showing over 0.99 AUC, indicating excellent separation of signal and noise. ***E***, UMAP projection of test dataset classifications showing spatial representation of final sEPSC model capacity to differentiate between noise and signal traces in the test dataset. ***F***, ***G***, Training validation accuracy and loss per epoch, showing sIPSC model training per iteration. ***H***, ROC curve for sIPSC detecting model, tested on 20% hold-out training data, showing over 0.99 AUC, indicating excellent separation of signal and noise traces. ***I***, UMAP projection of test dataset classifications showing spatial representation of final sIPSC model's capacity to separate signal from noise. Example training data shown in Extended Data Figure 2-1. Biological context of for training data shown in Extended Data Figure 2-2.

Each model was trained with miniML's open framework using a convolutional neural network (CNN) architecture optimized via hyperparameter tuning to maximize classification performance. Models were trained for up to 100 epochs using a binary cross-entropy loss function with an Adam optimizer. Training performance was evaluated on a held-out validation set representing 20% of the training data, tracking both classification accuracy and loss over training epochs (Fig. 2). Receiver operating characteristic (ROC) curves were generated from the final validation sets, with area under the curve (AUC) exceeding 0.99 for both sEPSC and sIPSC models, indicating near-perfect classification capability.

10.1523/ENEURO.0410-25.2026.f2-1 Figure 2-1 **Example signal and noise data used for training deep learning model** **(A)** Representative sIPSC training data recording. **(B)** Representative sEPSC training data recording. **(C)** Representative noise traces used for sIPSC / sEPSC model training. **(D)** Representative sample of individual hand-verified events and noise frames used for sIPSC model training (N = 200, N = 200). **(E)** Representative sample of individual hand-verified events and noise frames used for sEPSC model training (N = 200, N = 200). Download Figure 2-1, TIF file.

10.1523/ENEURO.0410-25.2026.f2-2 Figure 2-2 **Biological Context** Table showing biological context information such as strain, sex, age, region, and associated sample sizes for each study. Download Figure 2-2, CSV file.

To further demonstrate model discriminability, we projected the final-layer feature embeddings of the test data using Uniform Manifold Approximation and Projection (UMAP). The resulting low-dimensional plots showed clean separation between event and nonevent classes (Fig. 2), supporting the model's ability to encode physiologically relevant distinctions in the withheld portion of the training data. It is important to note that evaluating training performance in a held-out portion of the training data, while useful for model training supervision purposes, is not true evaluation of the detector in a new dataset since the held-out training data was compiled and hand verified from the same initial recordings.

### Deep learning model demonstration in unseen external collaborator dataset

In our testing on our own experimental data, the machine learning approach yielded impressive results compared with the other analysis rivaling a secondary hand-counter’s agreement to our primary hand-counted ground-truth analysis. We also qualitatively demonstrated use of the model in a small independent dataset collected by a separate research group using a different recording rig, brain region, and cell type (Fig. 5). The demonstration was run on two sIPSC and two sEPSC recordings from neurons in the olfactory bulb from female C57BL/6J mice (Extended Data Fig. 2-2).

Event detections from the model (MLD) and a secondary hand counter (HD2) were compared with hand-scored ground truth (Fig. 5). Precision and recall were evaluated across the entire test set, and *Fβ* scores (*β* = 0.3) were computed to qualitatively assess agreement with manual annotations (Fig. 5). To visualize detection across event magnitudes, events were binned by amplitude, and per-bin *Fβ* scores were calculated (Fig. 5). Overall, we see that both sIPSC and sEPSC performed similarly to that of the secondary hand counter as shown by their *Fβ* scores.

### General analysis and visualization methods

Data were exported from respective analysis pipelines and statistical analysis, and data visualization was performed in R-Studio v. 2024.12.1+563 (R version 4.4.0) or Visual Studio Code v. 1.107.1; Python version 3.10.13, using custom scripts. Final figure layouts were assembled using Adobe Illustrator v. 29.7.1. Analysis code was provided as Extended Data 1.

## Results

### Interlaboratory variability in synaptic event detection

While spontaneous event analysis has been done for decades, as far as we know there has never been a cross-lab validation study showing variance in sEPSC and sIPSC detection agreement between labs. Despite analyzing identical traces, individual lab's results varied significantly both in average amplitude and mean interevent interval (Fig. 1).

Experimenters differed significantly in how they classified sEPSC events (Fig. 1). The absolute value of the amplitude was used for easier visualization. Welch's ANOVA showed a significant effect of the experimenter on amplitude (Extended Data Fig. 1-1). Games–Howell post hoc comparisons revealed that Experimenter E reported significantly larger amplitudes than A, B, and D (Extended Data Fig. 1-1). No other pairwise differences in amplitude reached significance (Extended Data Fig. 1-1).

For sEPSC interevent intervals, Welch's ANOVA likewise indicated significant variation across annotators (Fig. 1; Extended Data Fig. 1-1). Games–Howell tests showed that Experimenter A reported longer IEIs than D but shorter IEIs than E (Extended Data Fig. 1-1). Experimenter B marked longer IEIs than D, while Experimenter D reported shorter IEIs than E (Extended Data Fig. 1-1). Other pairwise contrasts did not differ significantly (Extended Data Fig. 1-1).

There was substantial variability across annotators in both sIPSC amplitudes and interevent intervals (IEIs; Fig. 1). Welch's ANOVA showed a strong effect of the experimenter on amplitude (Extended Data Fig. 1-1). Games–Howell post hoc comparisons indicated that Experimenter B reported larger amplitudes than A, C, and D (Extended Data Fig. 1-1). Experimenter A reported larger amplitudes than C and D (Extended Data Fig. 1-1), while Experimenters C and D both reported smaller amplitudes than E (Extended Data Fig. 1-1). Other contrasts did not differ significantly (Extended Data Fig. 1-1).

For sIPSC interevent intervals, Welch's ANOVA again revealed significant differences among annotators (Fig. 1; Extended Data Fig. 1-1). Games–Howell tests showed that Experimenter A reported shorter IEIs than B and, C, but longer than D (Extended Data Fig. 1-1). Experimenter B reported longer IEIs than D and E (Extended Data Fig. 1-1). Similarly, Experimenter C reported longer IEIs than D and E (Extended Data Fig. 1-1). Finally, Experimenter D marked shorter IEIs than E (Extended Data Fig. 1-1). Other contrasts did not differ significantly (Extended Data Fig. 1-1).

Together, these analyses demonstrate systematic differences across labs in both the amplitude and IEI of sIPSCs and sEPSCs they considered events, underscoring variability in implicit detection thresholds between labs (Fig. 1; Extended Data Fig. 1-1).

### Benchmarking detector performance

**Figure 3.**
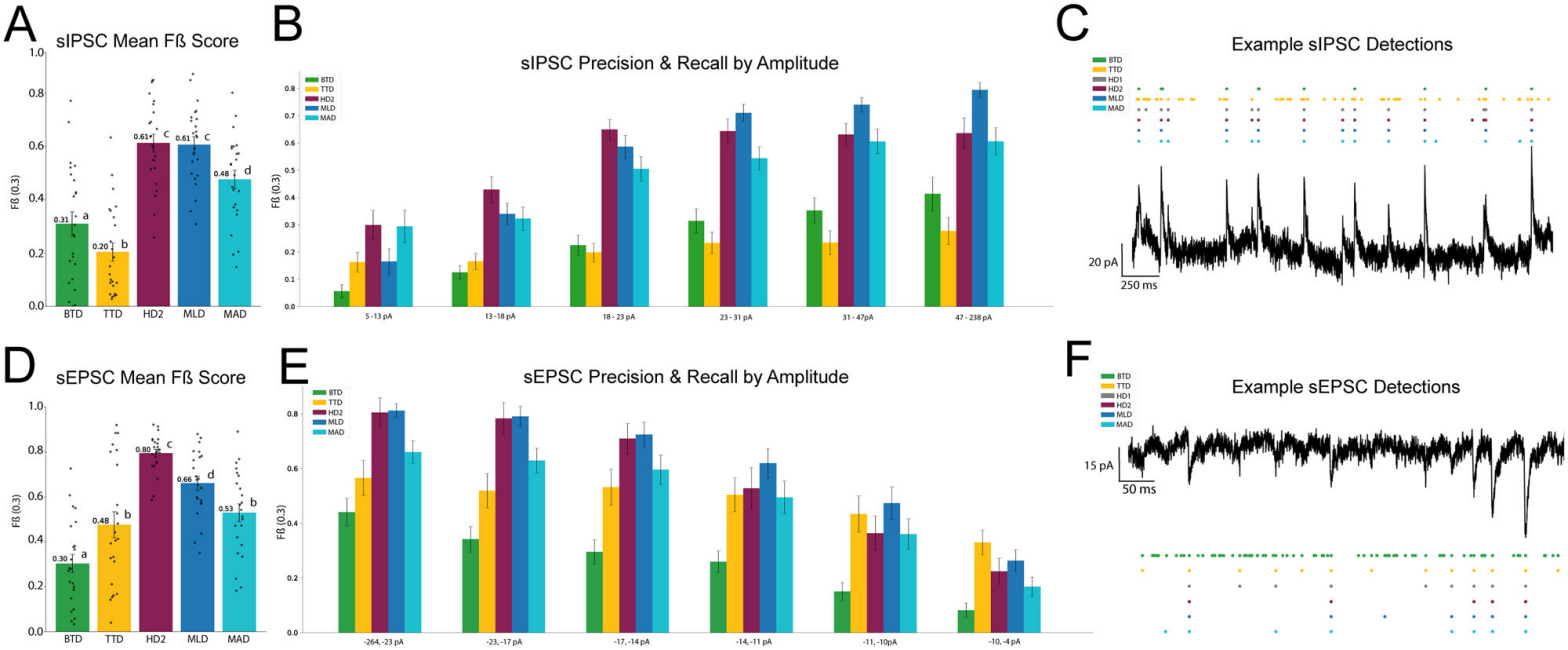
Benchmarking traditional sEPSC/sIPSC analysis methods and deep learning models. ***A***, Mean *F*-beta score benchmarking each detector's overall ability to detect sIPSCs against a hand-counted analysis (HD1). The machine learning model (MLD) achieves an *F*-beta score of 0.61, closely resembling the score of the secondary hand counter, and consistently outperforming traditional approaches. ***B***, *F*-beta scores calculated for each detector per amplitude range, across all analyzed sIPSC traces, showing progressive variance in detector accuracy for different sized events. ***C***, Example sIPSC detections for each detection approach. ***D***, *F*-beta score benchmarking each detector's overall ability to detect sEPSCs against a hand-counted analysis (HD1). The machine learning model (MLD) achieves an *F*-beta score of 0.66, consistently outperforming traditional approaches. A second hand counter (HD2) achieves an *F*-beta score of 0.80, showing high cohesion between hand counters in sEPSC data relative to automated detection methods. ***E***, *F*-beta scores calculated for each detector per amplitude range, across all analyzed sEPSC traces, showing progressive variance in detector accuracy for different sized events. ***F***, Example sEPSC detections for each detection approach. All error bars represent SEM. Full statistical reporting provided in Extended Data Figure 3-1.

Using a Gaussian generalized linear mixed-effects model with analysis method as a fixed effect and recording as a random intercept, sIPSC detector *Fβ* performance (relative to hand-counted standard) differed significantly across detection methods (Fig. 3; Extended Data Fig. 3-1). Holm-adjusted post hoc comparisons showed that HD2 significantly outperformed BTD, MAD, and TTD but did not differ from MLD. Alternatively, BTD performed significantly worse than MAD and MLD but outperformed TTD. MAD significantly underperforms MLD but outperforms TTD. MLD significantly outperforms TTD (Extended Data Fig. 3-1).

Using a Gaussian generalized linear mixed-effects model with analysis method as a fixed effect and recording as a random intercept, sEPSC detector *Fβ* performance (relative to hand-counted standard) differed significantly across detection methods (Fig. 3; Extended Data Fig. 3-1). Post hoc pairwise comparisons using Holm correction showed that HD2 outperforms each alternative detector. BTD showed significantly decreased performance compared with MAD, MLD, and TTD. MLD showed higher performance than MAD and TTD, whereas performance did not differ between MAD and TTD (Extended Data Fig. 3-1).

### Benchmarking detector sensitivity in pharmacological study

To assess whether detected synaptic events captured biologically relevant treatment effects, we evaluated each detection method's sensitivity to synaptic activity changes induced by systemic ethanol exposure prior to recording. Inhibitory and excitatory currents were recorded under voltage clamp from CRF+ neurons in the central amygdala.

**Figure 4.**
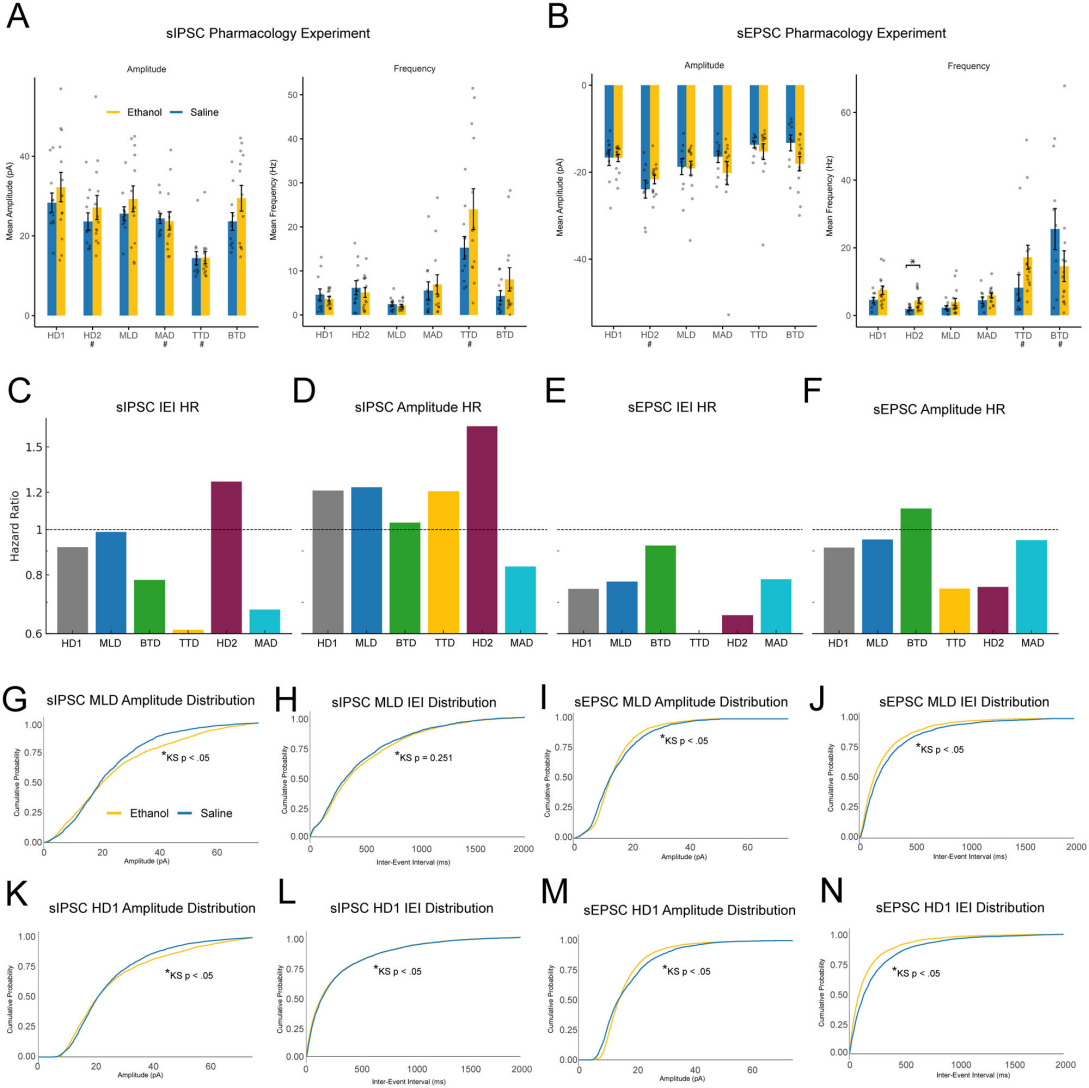
sEPSC and sIPSC detection results in a real-world pharmacology experiment. ***A***, Overall sIPSC frequency and amplitude results in an ethanol administration study showing high overall variance, but relative agreement in null-result outcome of the experiment across detection methods. Error bars represent SEM. ***B***, Overall sEPSC frequency and amplitude results in an ethanol administration study showing high overall variance, but relative agreement in null-result experimental outcome across detection methods except for HD2 which found a small but significant increase in sEPSC frequency due to ethanol administration. ***C–F***, Hazard ratios showing magnitude and direction of observed pharmacological effect size found in the same experimental dataset by each detection method for sIPSC/sEPSC IEI and amplitude. ***G–N***, Machine learning detection (MLD) and hand-counted (HD1) amplitude and IEI distributions show similar pharmacologically induced changes in sIPSC and sEPSC datasets due to IP ethanol treatment. Full statistical reporting provided in Extended Data Figures 4-1–4-3.

For each detector, a Welch's *t* test was used to compare pharmacology treatment (ethanol vs saline) effects on frequency which showed a significant ethanol-associated increase only for sEPSC frequency in HD2 (Fig. 4; Extended Data Fig. 4-1). All other sEPSC frequency tests were not significant (Extended Data Fig. 4-1). For mean sIPSC frequency, no method yielded a significant effect of ethanol (Extended Data Fig. 4-1). Welch's *t* tests were also used to compare mean amplitude between treatments (ethanol vs saline) per detector, yielding no significantly different comparisons (Extended Data Fig. 4-1).

Generalized linear mixed-effects models were used to compare synaptic event frequency and amplitude across analysis methods while accounting for repeated measurements within recording files. Analyses were performed separately for sEPSCs and sIPSCs. For each outcome (frequency or amplitude), we fit Gaussian GLMMs with AnalysisMethod as a fixed effect and recording as a random intercept, using HD1 as the reference level so that fixed-effect coefficients represent mean differences relative to HD1 on the raw scale (Fig. 4). Model estimates, standard errors, *z* statistics, and *p* values are reported in a supplementary table (Extended Data Fig. 4-2).

Empirical cumulative distribution functions (ECDFs) of detected event amplitude and IEI were generated, and group differences were assessed using two-sample Kolmogorov–Smirnov (KS) tests (Fig. 4; Extended Data Fig. 4-3). To quantify sensitivity to treatment effects, we conducted survival analysis by estimating Kaplan–Meier curves and fit Cox proportional hazard models to calculate hazard ratios for interevent interval (IEI) and amplitude distributions for each detector (Fig. 4; Extended Data Fig. 4-3).

Kolmogorov–Smirnov tests on the hand-counted detection revealed significant distributional differences between saline- and ethanol-treated groups across all synaptic measures. For sEPSCs, amplitude distributions differed between groups, with ethanol associated with a modest leftward shift. sEPSC interevent intervals were also strongly altered, reflecting longer IEIs under ethanol. For sIPSCs, amplitude distributions differed significantly, with ethanol producing larger amplitudes. sIPSC IEIs showed a smaller but significant divergence, with ethanol again associated with longer intervals (Extended Data Fig. 4-3). Kolmogorov–Smirnov tests on the machine learning detection dataset revealed significant distributional differences between saline- and ethanol-treated groups for most measures. For sEPSCs, amplitude distributions differed modestly, with ethanol being associated with a small leftward shift. sEPSC interevent intervals showed a strong divergence, reflecting longer IEIs under ethanol. For sIPSCs, amplitude distributions differed significantly, with ethanol producing larger amplitudes. In contrast, sIPSC IEIs did not differ significantly between groups (Extended Data Fig. 4-3).

## Discussion

Accurate measurement of synaptic transmission is foundational to basic neuroscience, providing integral mechanistic insight for brain-related drug discovery. Synaptic event detection methods have evolved significantly over the years, though the necessary balance between efficiency and accuracy has not always been effectively benchmarked. Reproducibility of electrophysiological results is contingent on accurate detection and subsequently high-quality benchmarking of detection methods. Recent work has highlighted the limitations of synaptic event detection and interpretation (Greger and Watson, 2024), and more modern approaches involving deep learning techniques have surfaced as potential options. The purpose of this work is to increase the consistency and replicability of synaptic event detection within the field by benchmarking commonly used detection techniques to a hand-verified ground truth.

To this end, we sought to first quantify the variance in detection between electrophysiology labs (Fig. 1) by asking multiple labs to apply their preferred analysis methods to the same sIPSC and sEPSC traces. Our findings show a lack of cohesion between scores, undermining the perceived reliability of current detection methods used by modern labs. In this initial evaluation of variability in the field, sEPSC and sIPSC recordings scored by different electrophysiology laboratories yielded significantly different results. The results indicate a range of identified event amplitudes and event frequencies (Fig. 1), suggesting the field does not have a unified understanding of how events are to be scored or at minimum that our lack of convergent scoring techniques leaves room for variability. The distributions clearly illustrate that experimenters from different labs are drawing their inclusion thresholds differently. Minimizing such variability between labs is paramount to the integrity of electrophysiology results going forward.

Our primary benchmarking analysis found that a secondary hand counter had the highest agreement with our primary hand counted ground-truth detection, but out of the automated detection methods tested, the deep learning model (MLD) and mean absolute deviation based detector (MAD) performed closest to our secondary hand counter, suggesting that more modern approaches to scoring sIPSC and sEPSC recordings can better replicate expert hand counts especially when compared with more basic threshold-based and template-based approaches (Fig. 3). We also demonstrate the ability to adapt the deep learning model to a small dataset collected from another electrophysiology lab. However, given the high potential for recording environment differences across labs, we suggest that other labs wishing to use this method take our process and model as a starting point and consider training their own model which may have the best potential of capturing lab-specific signal and noise signatures. While machine learning is a powerful tool, it is important to recognize its limitations, and further lab-specific, region-specific, and cell-specific validation is necessary. We hope our contribution here inspires other labs to conduct more validation of different event detection methods available on their own datasets, and in their own subdisciplines, which over time will allow the field to better improve upon analysis techniques and increase overall data quality within the field.

Benchmarking across detectors we can see how each automated detection method we performed on our own dataset compares to our hand-counted detection. Commonly used approaches such as BTD and TTD exhibited decreased precision and recall when compared with our practical ground-truth count. Conversely, the MLD strongly rivals the performance of our secondary hand counter overall. Next is the MAD technique, which outperforms the other detectors but overall falls behind the machine learning approach in our own dataset. Across all detectors, we see decreasing agreement with the practical ground truth as amplitudes get smaller both in sEPSC and sIPSC detection. This is not surprising as smaller amplitude events are inherently closer to the noise band and are by nature more difficult to detect regardless of the method used. It should be noted that even a secondary hand counter struggled to capture the same events as our practical ground truth, suggesting that even meticulous hand counting is highly variable for smaller amplitude events (Fig. 3).

10.1523/ENEURO.0410-25.2026.f3-1 Figure 3-1 **Statistical Results** Table showing GLMM and Holm corrected post hoc results comparing mean F-beta scores between detectors for sIPSC and sEPSC benchmarking. Download Figure 3-1, CSV file.

10.1523/ENEURO.0410-25.2026.d1 Data 1 **Analysis Code** Analysis Code, License, and Readme for all experimental results. Download Data 1, ZIP file.

Our comparisons of detection pipelines reveal relative congruence when looking at the mean differences between treatment groups for sEPSC and sIPSC frequency and amplitude in our pharmacology manipulation (Fig. 4). We used our practical ground truth (HD1) findings as a reference for evaluating the performance of the other pipelines accordingly. Our HD1 detection found no significant difference between conditions in mean amplitude or frequency in our sEPSC or sIPSC dataset. All but one other detection pipeline showed the same conclusion at the level of the mean. However, there was considerable variability in the overall calculated frequencies and amplitudes across detector pipelines when compared with the primary hand-counted dataset (Fig. 4). If we take our ground-truth comparison literally, this yields the finding that a pipeline that can find 3–4× as many events (interpreted then strictly as noise) as our HD1 pipeline will in general still find the same null result in mean difference between experimental groups. Perhaps equal noise is found between groups, so the signal remains relatively undistorted.

10.1523/ENEURO.0410-25.2026.f4-1 Figure 4-1 **Statistical Results** Table showing Welch corrected t-test results for sIPSC / sEPSC mean amplitude and frequency treatment comparisons for each detector. Download Figure 4-1, CSV file.

10.1523/ENEURO.0410-25.2026.f4-2 Figure 4-2 **Statistical Results** Table showing GLMM results comparing mean sIPSC / sEPSC amplitude and frequency for each detection method to reference group HD1. Download Figure 4-2, CSV file.

10.1523/ENEURO.0410-25.2026.f4-3 Figure 4-3 **Statistical Results** Table showing Kolmogorov–Smirnov tests comparing amplitude and frequency distribution histograms between ethanol and saline treatments for MLD and HD1 detection methods. Download Figure 4-3, CSV file.

Where mean population differences found between detection pipelines were largely the same in direction and magnitude, the more traditional version of synaptic event analysis relies on the generation of frequency distribution histograms which can more properly illustrate the shape of the data. They also allow for the interpretation of interevent intervals, which more directly reflect the timing of event arrival at the recorded cell than the raw frequency measures. In these comparisons, we found that while mean values were largely congruent, the frequency distribution histograms were not (Fig. 4). To further analyze the shifts in population, we then modeled our frequency distributions into probability-based extinction analysis so that effect sizes could be measured for the treatment of ethanol. In this way, we modeled the effect of alcohol as a function of the probability of experiencing either an amplitude or an IEI greater than the preceding point. This allowed for a comparison of the populations producing a hazard ratio (HR) by Cox regression so that the effect of alcohol treatment could be quantified instead of inferred visually after a KS test which is more standard. In this way, we have tabulated the calculated HRs across all detection pipelines to infer a magnitude of effect of alcohol treatment on sEPSC amplitude or IEI, or sIPSC amplitude or IEI and the results are striking. The MLD scores most closely follow our hand-counted values (Fig. 4), suggesting that the MLD did not simply find the same mean differences between populations but likely found a similar population of events to that of the expert hand scorer.

There is an unavoidable artifact within this project that should be discussed: our expert hand counter was the person who trained the curator of the MLD training dataset in electrophysiology, and therefore the reported success of the MLD can either be interpreted as evidence of the deep learning approaches merit or that the MLD inherited the scoring bias of expert hand counter by proxy. As an absolute ground truth is unknowable, we can state that the MLD produced scores most similar to the expert hand counter in our dataset, but we wish to highlight that neither the MLD nor our practical ground-truth detection can be considered infallible in this context. Which leads us to our next point: humans are variable. While our expert hand counter constrained their scoring sessions to limit variability as much as possible, it cannot be fully eliminated, and indeed our study indicates that even different hand scorers produce substantially varied results. Automated methods such as the MLD, however, do not tire of counting events, which in this instance may significantly reduce intraexperimenter data variability. This is where the implementation of a well-trained model with evaluated fidelity to an expert hand scorer may be especially valuable.

Also importantly, the present study focuses on synaptic event detection accuracy in the context of broad metrics such as event amplitude and frequency, which are some of the most commonly used in the field, but it does not probe more granular kinetics metrics as a biological endpoint. While reliable general kinetic features are necessarily leveraged implicitly or explicitly by any detection approach, measuring them accurately as a biological endpoint represents a more granular problem. Future studies are needed to explicitly validate event kinetics as a biological endpoint and should include pharmacological manipulations of event kinetics to probe the accuracy of granular kinetics measurements derived from automated detection pipelines.

**Figure 5.**
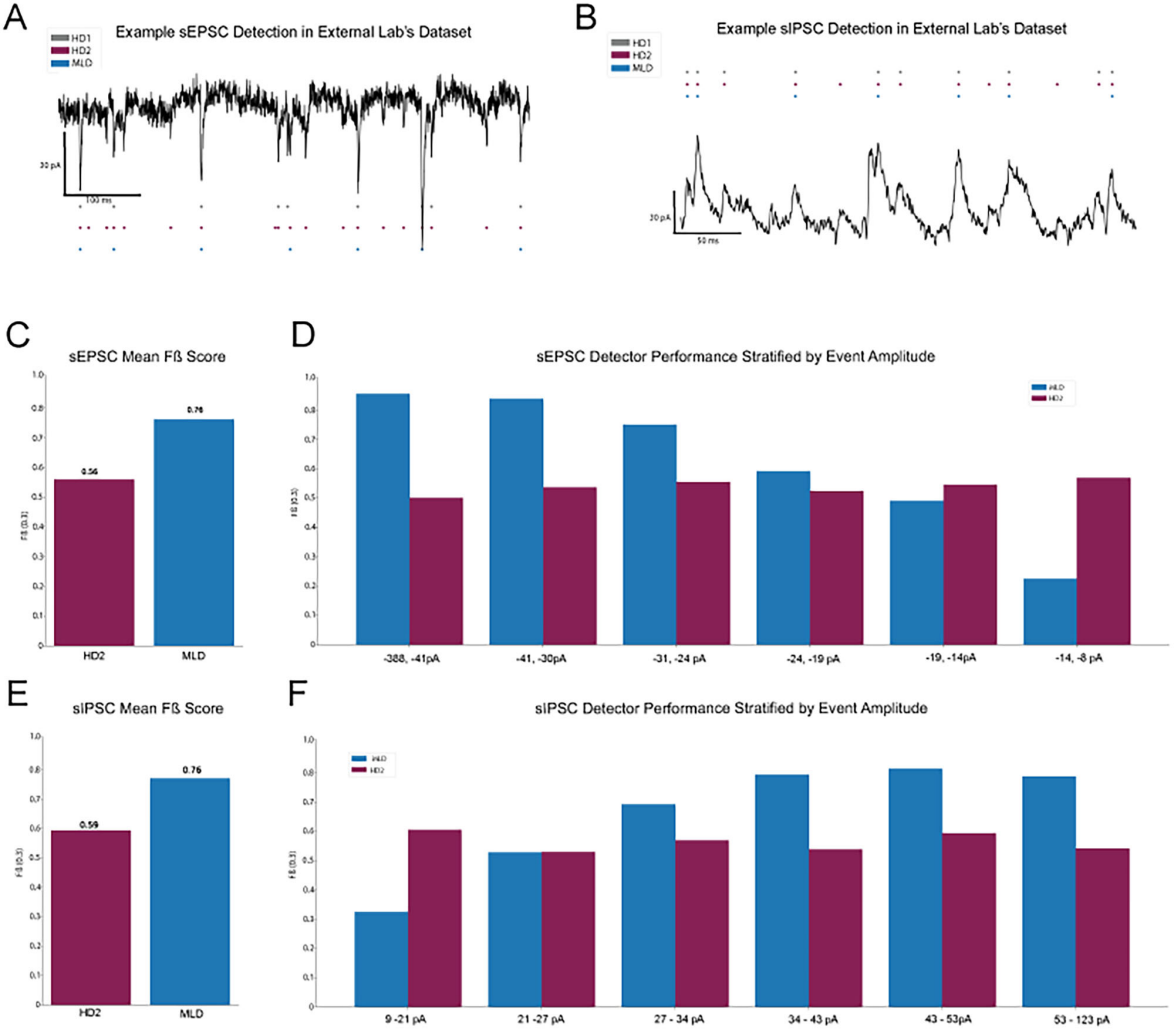
Deep learning trained detector demonstration on external collaborator sIPSC and sEPSC dataset. ***A***, ***B***, Example traces and detections on external collaborator sEPSC and sIPSC data, collected from neurons in the olfactory bulb. ***C***, ***D***, Overall and amplitude stratified *F*-beta scores demonstrating MLD performance and a secondary hand-counters performance on small external collaborator sEPSC recording dataset. ***E***, ***F***, Overall and amplitude stratified *F*-beta scores demonstrating MLD performance and a secondary hand-counters performance on small external collaborator sIPSC recording dataset.

In conclusion, accurate detection of synaptic events is imperative for mechanistic inquiry in neuroscience. Detection methods and analysis pipelines have grown in number and the need for evaluation within and across labs is vital. Our study demonstrates high variability between synaptic event detection methods across labs and in rigorous benchmarking on our own data. While we believe a hand-counted curation of synaptic physiology data to be the most robust method of scoring, we recognize the investment of time required and the potential for human-introduced variability in the process. We demonstrate that a deep learning model is capable of scoring a large, diverse, biologically relevant dataset with accuracy approaching that of a human expert's hand count. We also qualitatively demonstrate that the model has potential to be used in data generated by another lab from another cell type and region of interest (Fig. 5). While this causes us to be optimistic about the future of applying deep learning to synaptic physiology, we are not here to suggest blindly trusting such models nor do we believe we have made the only viable model in this space. Instead, we primarily want to encourage other labs to validate their detection methods seriously. We suggest that training a new model or adapting a model generated by another lab may ultimately lead to a higher quality detection method that may rival hand counting. We also posit that a combination approach, where an initial automated analysis is used, but some amount of hand verification is conducted as a second pass, may be a reasonable compromise for many labs. Our hope is that this work will encourage a future where synaptic event detection is more congruent between labs through higher benchmarking standards for event detectors and openness to continual validation of new detection methods as they evolve.

## Synthesis

Reviewing Editor: Adrien Peyrache, McGill University Decisions are customarily a result of the Reviewing Editor and the peer reviewers coming together and discussing their recommendations until a consensus is reached. When revisions are invited, a fact-based synthesis statement explaining their decision and outlining what is needed to prepare a revision will be listed below. The following reviewer(s) agreed to reveal their identity: NONE. Note: If this manuscript was transferred from JNeurosci and a decision was made to accept the manuscript without peer review, a synthesis may not be available.

This manuscript was reviewed by two experts in the field. Both felt that the data are relevant and robust, and the methodology for analyzing spontaneous events could be of benefit to the field. Specifically, it was noted that the manuscript highlights the variability in analysis between laboratories and train a deep learning model based on data from neurons in the ventral tegmental area (VTA), bed nucleus of the stria terminal (vBNST), and the central amygdala (CeA). The authors compare the deep learning model to other common analysis methods while recording from CRF+ neurons in the central amygdala (CeA) following an ethanol challenge. Finally, the authors compare the deep learning model to hand scoring on electrophysiological recordings in the olfactory bulb collected from another laboratory. While the findings are of interest, the following concerns and comments need to be addressed for a revision:

1. Provide the code used in these studies as supplementary material.

2. Per eNeuro policy, full, detailed statistical reporting needs to be included in a table. Further, some figures denote statistical significance with an asterisk, but most do not. (see other figure issues below)

3. Full reporting of key experimental details (i.e., cell number, animal sex, age, electrophysiological properties) needs to be included for all studies.

4. Justification for experimental design needs to be better described (i.e., regions used to train deep learning model, ethanol challenge). It is good to see the authors addressed noise variability in sEPSC/IPSC recordings, and that they accounted for that when training their deep learning model. However, since there is substantial variability in sEPSC/IPSCs (frequency, amplitude, kinetics) across brain region, sex, and animal strain, I am having trouble reconciling why the deep learning model was trained with data from only 3 brain regions (VTA, vBNST, CeA) with predominantly two different cell types (dopaminergic, GABAergic) in 1 strain (C57s). While the authors briefly mentions this in the methods section, additional context should be added to the discussion on why these specific regions were chosen, and how that might impact the utility of the deep learning model when analyzing data from regions with vastly different electrophysiological profiles.

5. While not often the focus of sIPSC/sEPSC experiments, the kinetics of the response are also important endpoints for studies in synaptic function. It would be beneficial to include this in the analytical workflow. In addition, I do not think that the authors need to do this, but a positive control that alters kinetics, such as benzodiazepines for IPSCs would have been a nice addition, potentially in future as a discussion point.

6. There was a recent preprint on mini analysis that is germane to this manuscript. Please read and provide some discussion.

https://www.biorxiv.org/content/10.1101/2024.10.26.620084v1

7. Methods, Access resistance: "RA was monitored throughout the recording and cells exceeding RA of 25 megaohms were not used in analysis." Please define RA as access resistance. Please justify why you included cells with a change in access resistance greater than 20%?

8. Figure 1: There are no asterisks to indicate the statistical significance as discussed in the results section.

Figure 1: Please include justification for recording in the olfactory bulb for the sIPSC and sEPSC traces that were analyzed by different laboratories.

Figure 1: The authors should include sEPSC and sIPSC kinetics data (i.e., rise and decay) across experimenters.

Figure 1A,B,D,E: The methods section states that each experimenter was "sent the same sEPSC and one sIPSC trace" to analyze, so it is unclear what the average and SEM in each of the graphs represents. If the data was binned and averaged within a single recording, please specify and add the individual data points to the bar graphs.

Figure 1A,B,D,E: Please make the colors consistent among all graphs. In Figures 1A and 1B experimenter D is blue, whereas in Figures 1D and 1E experimenter D is green.

Figure 1G,I: It is unclear why there is an experimenter F in these graphs, but not in the previous graphs in this figure.

Figure 1A,B,G,H: There is no explanation for the lack of data from Experimenter C or E.

Figure 1G,H,I,J: There is no mention of this data in the results section, or whether there are statistically significant differences between groups.

Figure 1C,F: The x-axis is labelled as seconds and based on the y-axis from Figures 1B and 1E, it should be msec.

9. Figure 2: There are no statistical findings for data in the results section.

Figure 2: Please include representative traces of the variety of noise that you used to train the model. Please also include representative traces or any form of graphical representation from sIPSC/EPSC recordings to demonstrate the distribution of events used to train the model.

Figure 2: There is no mention of whether collection of sEPSC and sIPSC slice electrophysiology recordings to train the model came from male and/or female C57Bl/6 mice.

Figure 2A: The number of cells, brain regions, sexes, and rodent strain should be included in the "Compile Training data" section of the schematic. The schematic itself is vague, so a brief summary of each step (1-2 bullet points) will be more useful for the reader.

10. Figure 3A,B,D,E: The labels on the axes of these graphs are grainy and difficult to read.

Figure 3E: The y-axis is unlabeled and there is no line for the x-axis, as in Figure 3B.

11. Figure 4: Please add justification for recording from CRF+ CeA neurons in the manuscript.

Figure 4: The authors should include sEPSC and sIPSC kinetics data (i.e., rise and decay) in Figure 4A and 4B.

Figure 4: Please include the average membrane properties (i.e., capacitance, membrane resistance, access resistance) of the cells recorded, the number of animals (and sex of those animals) recorded in each treatment group, and the age range of the animals when recorded.

Figure 4: It would be helpful for the authors to measure and report the blood alcohol concentrations (BACs) age- and sex-matched to the animals used for electrophysiological recordings following a similar experimental timeline (60 minutes following a 2g/kg ethanol injection). And provide rationale for why they chose this experiment as the pharmacological manipulation.

Figure 4A: There are no asterisks to indicate the statistical significance as discussed in the results section.

Figure 4G-N: It is unclear what the "ML EA" "ML EI" "GT EA" and "GT EI" labels on the graphs mean. Some of the graphs do not have y-axes (Fig. 4I, K, M). Graphs are grainy when zoomed in. The units for amplitude should also be "pA" and not "Pa."

12. Figure 5: Authors state HD2 and MLD analysis is comparable, but they do not provide statistical analysis of the Fβ scores in the results section.

Figure 5: The method section for Figure 5 mentioned that this dataset included recordings from male and female mice but did not specify what strain. Please include the average membrane properties (i.e., capacitance, membrane resistance, access resistance) of the cells recorded, the number of cells/animals (and sex of those animals), and the age range of the animals when recorded.

## Author Response

RESPONSE TO THE REVIEWERS We are grateful for the time and care invested by the editor and reviewers in evaluating our manuscript and the provision of constructive feedback which we feel has strengthened its clarity and rigor. We have addressed each comment and appropriately revised the text (revisions highlighted in yellow throughout the main text) and figures to reflect the feedback resulting in a stronger, clearer manuscript.

In response we have made several major revisions and additions including the provision of all analysis code. In accordance with eNeuro's policy we have moved our statistical reporting to supplementary tables and included a table outlining biological context such as animal strain, sex, age, and cell numbers across experiments. In conjunction we have revised the methods and discussion sections to justify experimental design choices as requested.

We valued the request for examples of data used to train the deep learning model and have included a new supplementary figure with example traces and specifically example noise used in the training process.

We are also grateful for the thorough review and feedback regarding our figures and have updated them to reflect the suggestions and requests, as outlined line by line below.

We believe these revisions fully address the reviewers' concerns and sharpen the manuscript overall. Our full responses are outlined line by line below. Thank you again for the opportunity to revise and resubmit this work and we look forward to your response.

1. Provide the code used in these studies as supplementary material.

We have provided the code used as requested in a Zenodo repo.

2. Per eNeuro policy, full, detailed statistical reporting needs to be included in a table.

Further, some figures denote statistical significance with an asterisk, but most do not. (see other figure issues below) We thank the reviewers for pointing out the eNeuro policy, and now statistical results have been summarized in supplementary tables and referenced in the results section. Also similarly statistical significance is now reported on the figures where appropriate. Thank you for alerting us to this eNeuro policy.

3. Full reporting of key experimental details (i.e., cell number, animal sex, age, electrophysiological properties) needs to be included for all studies.

We thank the reviewers for highlighting the need for clarity and full reporting of key experimental details.. We agree that biological context is important for future interpretation and have added Supplementary Table (Figure 2-2) to fully report animal strain, sex, age, and cell number for all studies. Regrettably, some electrophysiological properties such as membrane capacitance and membrane resistance were not available to report as we do not routinely record them when performing patch clamp studies on CRF+ neurons as we identify them by fluorescence reporting and not by cellular characteristics. Again, we appreciate the reviewers feedback here and believe the addition of supplementary table (Figure 2-2), as well as additional reporting in our methods section strengthen the future interpretability of the manuscript.

4. Justification for experimental design needs to be better described (i.e., regions used to train deep learning model, ethanol challenge). It is good to see the authors addressed noise variability in sEPSC/IPSC recordings, and that they accounted for that when training their deep learning model. However, since there is substantial variability in sEPSC/IPSCs (frequency, amplitude, kinetics) across brain region, sex, and animal strain, I am having trouble reconciling why the deep learning model was trained with data from only 3 brain regions (VTA, vBNST, CeA) with predominantly two different cell types (dopaminergic, GABAergic) in 1 strain (C57s). While the authors briefly mentions this in the methods section, additional context should be added to the discussion on why these specific regions were chosen, and how that might impact the utility of the deep learning model when analyzing data from regions with vastly different electrophysiological profiles.

We apologize for any lack of clarity regarding our chosen regions of training data and we have amended our discussion section with a larger discussion of what was included for training, and what kinds of limitations the training data could represent. To train this model, we used all available gap free recordings of sEPSCs and sIPSCs that we had generated in the last few years, as we believed a larger sampling of data from our rig across all possible data types would generate a model that could identify events well. While we agree that including more region and cell types is always better, but like most electrophysiology labs ours focuses on a few core regions of interest. To increase the diversity of brain regions and cell types in the training dataset, we included data from CeA, VTA, and BNST in heterogeneous cell populations. Our discussion section now reflects more appropriately the limitations of the models training, and we have also updated the discussion section to better reflect what we believe this model is appropriate for; that is using this model as an example and potential starting for generating high quality lab specific or even region specific models to account for variability of events and rig specific noise. We agree that both synaptic events and recording noise dynamics vary greatly across region, sex and strain. While in theory we are enthusiastic about the idea of training a model on a much further reaching dataset, and hope that may be possible some day, our primary goal was to generate and test the capacity of a model within the context of our own laboratory. One of our hypotheses as to why detection varies so greatly between labs is that recording practices, equipment at the slice level may introduce noise that is lab / rig specific.

Our goal was to use as much data from our own lab as possible to train a model capable of parsing particularities due to our specific recording procedures and setup. In doing so, we used all synaptic physiology data available for the project to train our model including recordings from (VTA, vBNST, CeA) in both C57 background mice as well as sprague dawley rats and long evens rats. In the initial manuscript we were heavily focused at the event level, but have now updated the manuscript to describe the full biological context of the training dataset, as well as the rationale including a supplementary figure (Figure2-2). Thank you for this valuable feedback.

5. While not often the focus of sIPSC/sEPSC experiments, the kinetics of the response are also important endpoints for studies in synaptic function. It would be beneficial to include this in the analytical workflow. In addition, I do not think that the authors need to do this, but a positive control that alters kinetics, such as benzodiazepines for IPSCs would have been a nice addition, potentially in future as a discussion point.

We want to thank the reviewer for this thoughtful suggestion and agree that kinetics are indeed important endpoints in many electrophysiological studies. The primary objective of the present work is to benchmark detection performance with a focus on detection accuracy, amplitude and event frequency which are commonly used metrics in our domain and themselves in need of validation and better detection fidelity. While we are also interested in detailed kinetics measurements, the present study is not designed to probe accurate kinetics measurements and substantial effort will be required to generate a ground truth dataset of high enough quality and granularity to validate kinetics measurements effectively. While we would like to explore deeper kinetics benchmarking in future work, it does not fall into the intended scope of the present paper. Ethanol itself is also a positive allosteric GABA modulator, though not as potent as benzodiazepines. Our ethanol manipulation does at least to some degree function as a test of how event dynamic alteration may affect detector differences. We agree that a benzodiazepine modulation as a kinetic control will be highly beneficial when we embark on future kinetics focused work and have added discourse about such necessary future work to our discussion section. Thank you for the valuable insight, we think it has greatly enhanced the scope of our discussion of future study potential.

6. There was a recent preprint on mini analysis that is germane to this manuscript. Please read and provide some discussion. https://www.biorxiv.org/content/10.1101/2024.10.26.620084v1 We thank the reviewers for drawing our attention to this valuable preprint, as its contents are highly relevant to our work. Within our introduction we have added discussion and reference to this paper.

7. Methods, Access resistance: "RA was monitored throughout the recording and cells exceeding RA of 25 megaohms were not used in analysis." Please define RA as access resistance. Please justify why you included cells with a change in access resistance greater than 20%? We thank the reviewers for this clarification question and attention to detail. We applied an absolute access resistance cutoff because voltage-clamp fidelity degrades nonlinearly as access resistance (RA) increases, and relative percent-change rules weight small, inconsequential changes at low Ra (change in 4 →6) the same as changes that meaningfully compromise control at higher Ra (Ra 20→ 25). Small shifts from very low Ra values have negligible impact on voltage control, whereas recordings that reach higher absolute Ra approach a regime where clamp speed and accuracy are unreliable even if the relative change is modest. An absolute cutoff therefore more directly enforces a minimum standard of voltage control across recordings.

8. Figure 1: There are no asterisks to indicate the statistical significance as discussed in the results section.

In order to avoid a difficult to follow tree of comparisons we added significance letters above each bar to improve readability.

9. Figure 1: Please include justification for recording in the olfactory bulb for the sIPSC and sEPSC traces that were analyzed by different laboratories.

We thank the reviewers for the opportunity to clarify the selection of the olfactory bulb. The olfactory bulb data is for the demonstration of the final ML model on a new type of data as an example suggesting that starting with a pretrained model may be viable in some situations with proper validation (Figure 5).

The Figure 1 data is a separate study and is meant to be a preliminary examination variance between current electrophysiology labs analysis of the same real IPSC and EPSC traces which were recorded in our lab from CeA CRF+ neurons. We have refined the explanation of these studies in the text and provided a supplementary table (Figure 2-2) outlining the recording datasets used for each study.

10. Figure 1: The authors should include sEPSC and sIPSC kinetics data (i.e., rise and decay) across experimenters.

We agree with the reviewers and believe that kinetics are an important measurement of the deeper biophysical aspects of synaptic events, however, the focus of our paper is on the identification of events in time and measurement of their amplitudes. While we believe this is a valuable direction for this project moving forward, it is beyond the scope of the project in its current form. For our interlab comparison we did not request full event kinetics nor stress the accuracy of such data and therefore have not included that. In our view, thorough and accurate kinetic measurement as a biological endpoint is exceedingly difficult. While many automated analysis methods are capable of producing individual event kinetics data, in our experience the results can be highly inaccurate. Many labs in our field use broad frequency and amplitude data and do not utilize high resolution kinetics data, so while we hope to eventually benchmark detectors for kinetics data, it will require hand verification of accurate kinetic measurements for every event in our practical ground truth dataset. We are excited about the prospect of future work in this domain as the scope of a future paper.

11. Figure 1A,B,D,E: The methods section states that each experimenter was "sent the same sEPSC and one sIPSC trace" to analyze, so it is unclear what the average and SEM in each of the graphs represents. If the data was binned and averaged within a single recording, please specify and add the individual data points to the bar graphs.

The average and SEM are based on all detected events, using individual events as N. The distributions in panels G - J visualize this in cleaner form than individual data points on the bar graph can due to the sheer number of events detected in a single trace. For this analysis, our goal was to show the variance in the amplitude and IEI of individual events detected by different labs in an identical trace. Another option might be to run a KS test and compare the distributions, but we fear that would be too sensitive an analysis, and do not have the ability to make any sort of specific hypothesis in this case.

12. Figure 1A,B,D,E: Please make the colors consistent among all graphs. In Figures 1A and 1B experimenter D is blue, whereas in Figures 1D and 1E experimenter D is green.

13. Figure 1G,I: It is unclear why there is an experimenter F in these graphs, but not in the previous graphs in this figure.

Thank you for pointing this out. We found the bug in our plotting script and have addressed the inconsistencies.

14. Figure 1A,B,G,H: There is no explanation for the lack of data from Experimenter C or E.

Experimenter C due to unknown technical reasons (collaborating lab) was unable to analyze the EPSC file, but did analyze the IPSC file. We therefore included what we could, but still wanted the experimenter group letters to correspond between EPSC and IPSC graphs. We have added a line to the methods section pertaining to this analysis in the manuscript explaining.

15. Figure 1G,H,I,J: There is no mention of this data in the results section, or whether there are statistically significant differences between groups.

We thank the reviewers for this observation. These are meant to show the distribution of individual events summarized in the earlier panels as individual datum points on the bar graphs would be very difficult to interpret due to the quantity of events detected by each experimenter. These are meant to show descriptively the distribution of amplitude and IEI found by each experimenter.

16. Figure 1C,F: The x-axis is labelled as seconds and based on the y-axis from Figures 1B and 1E, it should be msec.

We apologize for this mislabel and have corrected the figure.

17. Figure 2: There are no statistical findings for data in the results section.

Yes, we agree that the figure does not contain hypothesis testing statistics and thus discussion of it may not belong in the results section, but rather the methods section. This is an important and intentional distinction. One of the problems with training ML models is that within training validation as shown in this figure is flawed in that the validation occurs only in a subset of data derived from the training dataset. This is useful for descriptively showing validity of the training process itself, but it does not constitute a realworld test of performance.

Importantly, training datasets are intrinsically biased in their construction as they are generated specifically to approximate a particular notion of what ground truth should look like under a specific set of assumptions. For this reason our primary statistical analysis is performed on independently collected, whole datasets entirely separate from the training dataset. So, statistical analysis showing the models real-world performance is shown in figures 3 and 4 and their associated analysis tables (Figure 3-1, Figure 4-1, Figure 4-2, Figure 4-3).

We want to validate the observation that no hypothesis testing occurs in figure 2 as it is meant to describe and show internal validity of the training process, and point the reviewers to the following two figures which provide rigorous real world testing of the model against a human annotated dataset. As such we have moved discussion of figure 2 from the results section to the methods section where we think it fits better for the sake of clarity. While we believe this data maybe useful to those training their own models, it could be made supplementary, as it's intended to provide clarity to the model training process rather than itself be a benchmark of real-world performance.

18. Figure 2: Please include representative traces of the variety of noise that you used to train the model. Please also include representative traces or any form of graphical representation from sIPSC/EPSC recordings to demonstrate the distribution of events used to train the model.

We have included new panels with a sample of overlayed representative noise and signal traces from both IPSC and EPSC models in a new supplementary figure (Figure 2-1). It is important to note these are only a small subset of the much larger training datasets, but were randomly sampled for visualization purposes (n = 200) to show visually the variety of signal and noise contained in the datasets.

19. Figure 2: There is no mention of whether collection of sEPSC and sIPSC slice electrophysiology recordings to train the model came from male and/or female C57Bl/6 mice.

Thank you for pointing out this lack of clarity. As mentioned in a previous response the model was trained with the intention of including as much data specific to our lab and recording environment as possible and as such we included as many clear event and noise segments as possible. Biological context for the training dataset and our lab has been now included in the text and as a supplementary table (Figure 2-2).

20. Figure 2A: The number of cells, brain regions, sexes, and rodent strain should be included in the "Compile Training data" section of the schematic. The schematic itself is vague, so a brief summary of each step (1-2 bullet points) will be more useful for the reader.

We agree with the reviewers point that the schematics are undetailed and have changed their formatting to be bullet points and to contain the information suggested. Thank you for this feedback. As mentioned we have included region, sex, rodent information in methods and as a supplementary table (Figure 2-2).

21. Figure 3A,B,D,E: The labels on the axes of these graphs are grainy and difficult to read.

Figure 3E: The y-axis is unlabeled and there is no line for the x-axis, as in Figure 3B. Thank you for pointing this out, we have done our best to improve image quality readability of this figure.

22. Figure 4: Please add justification for recording from CRF+ CeA neurons in the manuscript.

Thank you for this suggestion. Our goal was to test detectors in a highly constrained study of actual interest to our lab. CRF+ CeA neurons are known to be important in ethanol seeking behavior and are a highly homogenous population, allowing us to benchmark detectors within an actual study of interest in a controlled population of cells. This rationale has been added to our methods section, and we thank you again for this suggestion.

23. Figure 4: The authors should include sEPSC and sIPSC kinetics data (i.e., rise and decay) in Figure 4A and 4B.

We agree that kinetics are often an important biological output for electrophysiology studies, however the current study is focused on validating more basic metrics of frequency and amplitude. In future work we hope to generate a thorough ground truth dataset capable of benchmarking different detector's more granular kinetics outputs, but this is beyond the scope of the current paper. We have added a description of the importance of future work in this area to our discussion and thank the reviewers for pointing out this important next step.

24. Figure 4: Please include the average membrane properties (i.e., capacitance, membrane resistance, access resistance) of the cells recorded, the number of animals (and sex of those animals) recorded in each treatment group, and the age range of the animals when recorded.

Thank you for bringing up these important points. Animal experimental details have been added to the text. Access resistance for all cells was monitored during recordings and maintained below 25 megaohms as outlined in the electrophysiology methods. Capacitance and membrane resistance were not routinely recorded due to the highly constrained population of CeA CRF+ neurons which were transgenically labeled, and do not differ greatly in cell type nor size. Importantly comparisons were performed within this constrained population using identical recording configurations and inclusion criteria.

25. Figure 4: It would be helpful for the authors to measure and report the blood alcohol concentrations (BACs) age- and sex-matched to the animals used for electrophysiological recordings following a similar experimental timeline (60 minutes following a 2g/kg ethanol injection). And provide rationale for why they chose this experiment as the pharmacological manipulation.

We agree this would be beneficial to the pharmacology study here, though BAC's were not collected in the study at hand and we did not deem necessary for the scope of the paper which is focused on reliability of event detection. BAC's using this dose have been shown in mice previously: https://pmc.ncbi.nlm.nih.gov/articles/PMC3527904/. We thank the reviewers for this important point and have added reference to this in the pharmacology study section of our methods.

26. Figure 4A: There are no asterisks to indicate the statistical significance as discussed in the results section.

We have added asterisks where applicable and full statistical reporting has been provided as requested in supplementary tables (Figure 4-2, Figure 4-3). Thank you for this observation.

27. Figure 4G-N: It is unclear what the "ML EA" "ML EI" "GT EA" and "GT EI" labels on the graphs mean. Some of the graphs do not have y-axes (Fig. 4I, K, M). Graphs are grainy when zoomed in. The units for amplitude should also be "pA" and not "Pa." We have improved the overall legibility of these figures, updating the labels to better match the rest of the paper (MLD, HD1). We have also done our best to increase the fidelity of the figures. Thank you for these notes.

28. Figure 5: Authors state HD2 and MLD analysis is comparable, but they do not provide statistical analysis of the Fβ scores in the results section.

We purposefully refrained from testing a full dataset due to the massive amount of work it would take for two handcounters to thoroughly generate practical ground truth data, and due to the extreme amount of data we would need to request from our collaborators for this specific purpose.

Our intent was to show how well a locally trained model works on data from another labs recording environment and region of interest. We do not however want to give the impression that a model trained in one lab can be fully generalizeable, as we are skeptical of that possibility in the first place. As mentioned previously by a reviewer, our training dataset is limited to our own rig and ROI's. So the more informative test is the thorough examination of the model's performance on our own dataset for which it was intended.

The goal of our study is to highlight the difficulty of event detection, the variation even amongst trained hand counters, and the importance of within-lab validation of any detection technique. Due to the lack of hypothesis testing and the demonstrative nature of Figure 5, we have moved text from the results section to the methods section of the paper, and added brief discussion of the response outlined here to our discussion section.

29. Figure 5: The method section for Figure 5 mentioned that this dataset included recordings from male and female mice but did not specify what strain. Please include the average membrane properties (i.e., capacitance, membrane resistance, access resistance) of the cells recorded, the number of cells/animals (and sex of those animals), and the age range of the animals when recorded.

This figure is intended as a qualitative demonstration of how the model trained in our lab might be used as a starting point for other labs. In this interest we procured from a collaborating lab, 2 sIPSC and 2 sEPSC recordings from female 7 week old mice. We have included this information in a supplementary table (Figure 2-2).
