# Supplementary figures and images for: Optimizing and Benchmarking Machine Learning and Traditional Synaptic Event Detection Pipelines in Neurophysiology Experiments
Source: eNeuro. 2026 Apr 29;13(5):ENEURO.0410-25.2026. doi: 10.1523/ENEURO.0410-25.2026 (PMC13233908; doi:10.1523/ENEURO.0410-25.2026)

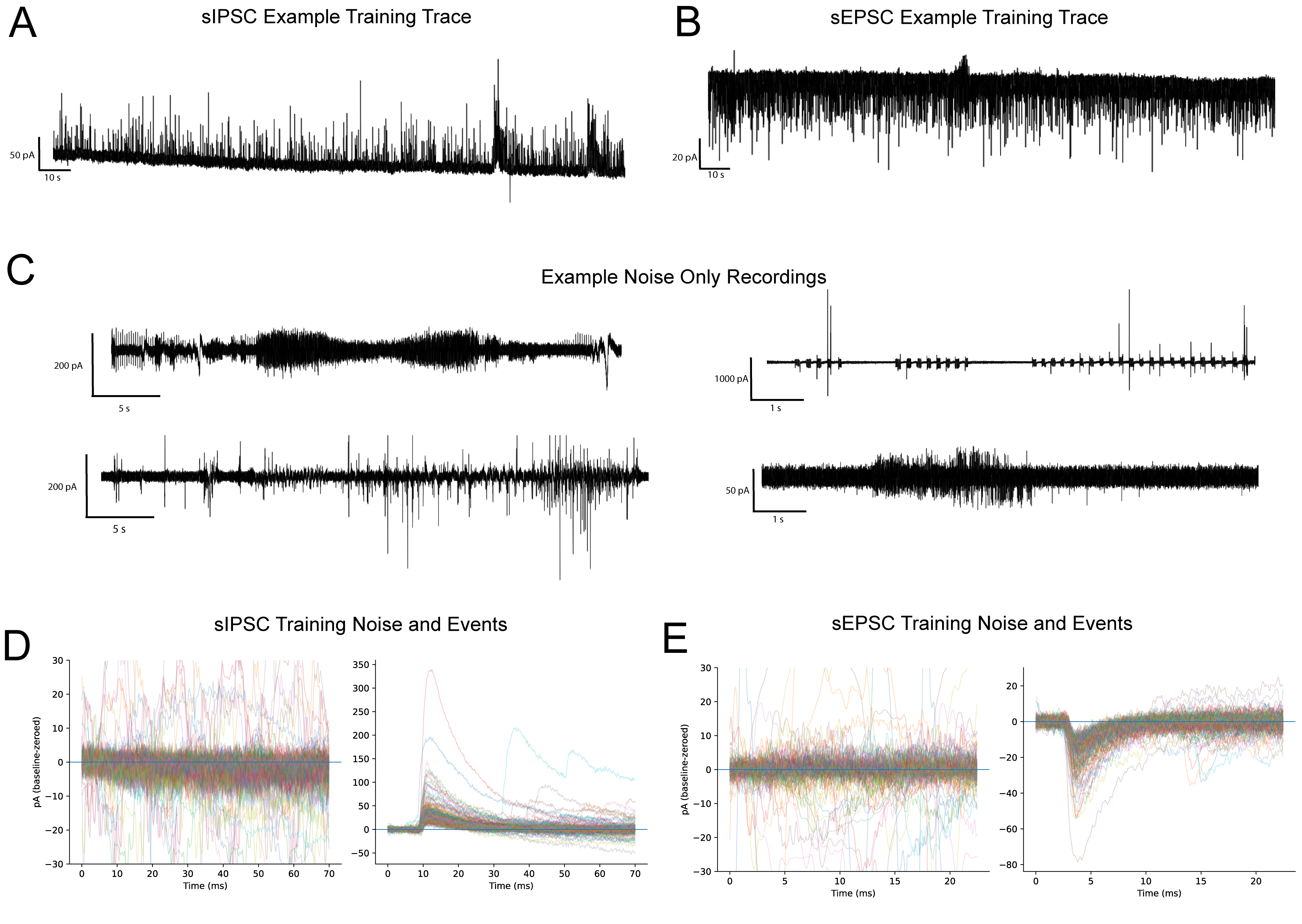

Supplement: Figure 2-1 — Example signal and noise data used for training deep learning model (A) Representative sIPSC training data recording. (B) Representative sEPSC training data recording. (C) Representative noise traces used for sIPSC / sEPSC model training. (D) Representative sample of individual hand-verified events and noise frames used for sIPSC model training (N = 200, N = 200). (E) Representative sample of individual hand-verified events and noise frames used for sEPSC model training (N = 200, N = 200). Download Figure 2-1, TIF file. [file eneuro-13-ENEURO.0410-25.2026-s002.tif]
